# Exploration of solid-state nanopores in characterizing reaction mixtures generated from a catalytic DNA assembly circuit†
†Electronic supplementary information (ESI) available. See DOI: 10.1039/c8sc04875d


**DOI:** 10.1039/c8sc04875d

**Published:** 2018-12-13

**Authors:** Zhentong Zhu, Ruiping Wu, Bingling Li

**Affiliations:** a State Key Lab of Electroanalytical Chemistry , Changchun Institute of Applied Chemistry , Chinese Academy of Science , Changchun , Jilin 130022 , P. R. China . Email: binglingli@ciac.ac.cn; b University of Chinese Academy of Sciences , Beijing , 100049 , China; c University of Science and Technology of China , Hefei , Anhui 230026 , China

## Abstract

Recent advances have proven that using solid-state nanopores is a promising single molecular technique to enrich the DNA assembly signaling library. Other than using them for distinguishing structures, here we innovatively adapt solid-state nanopores for use in analyzing assembly mixtures, which is usually a tougher task for either traditional characterization techniques or nanopores themselves. A trigger induced DNA step polymerization (SP-CHA), producing three-way-DNA concatemers, is designed as a model. Through counting and integrating the translocation-induced current block when each concatemer passes through a glass conical glass nanopore, we propose an electrophoresis-gel like, but homogeneous, quantitative method that can comprehensively profile the “base-pair distribution” of SP-CHA concatemer mixtures. Due to the higher sensitivity, a number of super long concatemers that were previously difficult to detect *via* gel electrophoresis are also revealed. These ultra-concatemers, longer than 2 kbp, could provide a much enhanced signal-to-noise ratio for nanopores and are thus believed to be more accurate indicators for the existence of a trigger, which may be of benefit for further applications, such as molecular machines or biosensors.

## Abstract 2

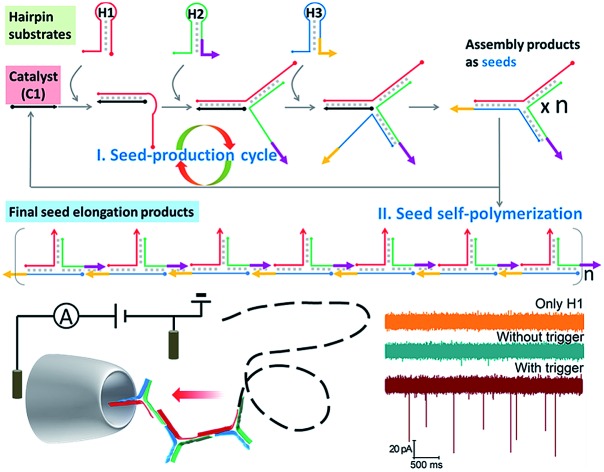
We adapt a solid-state nanopore for analyzing DNA assembly mixtures, which is usually a tougher task for either traditional characterization methods or nanopores themselves. A trigger induced nucleic acid amplifier, SP-CHA, is designed as a model. We propose an electrophoresis-gel like, but homogeneous, quantitative method that can comprehensively profile the “base-pair distribution” of SP-CHA concatemer mixtures.

## Introduction

From the disclosure of the nucleic acid double helix structure^1^ to the rapid development of DNA nanotechnology^2^–^10^ and computing,^11^–^22^ DNA has been engineered to be much more than genetic species, but rather to be a highly controllable “programmable material”, like LEGO bricks. Through the elaborate control of hybridization thermodynamics or kinetics, these bricks can be assembled into a variety of sophisticated structures^2^–^10^ or circuits^11^–^22^ for different potential applications,^2^–^36^ such as bio-computing, molecular machines, amplifiers, biosensors, disease diagnosis, and even cancer therapy.

Compared with the above quick successes in design and functionalization, the characterization methodologies for DNA assembly behaviours are relatively out of step.^37^ For example, other than various readouts (*e.g.*, FRET and electrochemistry) to sense assembly rates or amounts, gel electrophoresis and microscopy (*e.g.* AFM or TEM) are still a highly dependent "signal combo" to verify structure formation.^37^ Due to some inherent shortcomings, such as those related to surface scanning, strong electric fields, or low sensitivity, these commonly used techniques may not be fully enough to reveal accurate structural information, size distribution, or dynamic change data, especially in homogeneous solutions. On the whole, this may restrict a refined understanding of DNA assemblies and therefore affect their application efficiency. Therefore, the enrichment of detection methodologies is still an urgent demand.

In this research, we explored a single molecular sensing tactic, nanopore detection, for its possible use to profile DNA assembly at the single-molecule level and in homogeneous solutions. The basic principle of nanopore detection is that modulations in ionic current reflect the translocation of single macromolecules through a nanoscale aperture.^38^ After more than twenty years of development and improvement, it has been proven to be a powerful analysis platform that enables label-free and separation-free single-molecule analysis.^39^–^52^ For example, pores made of proteins (bio-pores) have shown extremely high resolution in analysing targets with small diameters.^53^–^58^ The most representative success is the invention of the 4th-generation of gene sequencing.^40^,^51^,^52^ For comparison, the other class of pores fabricated using solid-state materials (such as silicon nitride^59^–^65^) offer several potential points of superiority over bio-pores in terms of chemical, mechanical and thermal robustness and better flexibility for detecting huge target molecules, which could not pass through bio-pores. However, besides conventional shortcomings, such as more difficult fabrication and poorer reproducibility, a big challenge that still limits the application of solid-state nanopores is their lower resolution compared to bio-pores. Increasing efforts are being made to solve this problem, *e.g.* adjusting the nanopore geometry,^66^,^67^ using two-dimensional materials,^68^ building DNA origami nanopores,^69^–^71^ chemical modification,^49^,^50^,^72^–^77^ *etc.* Imperative data processing^78^,^79^ and novel strategies in which dynamic ionic flow was used instead of a classic “metal wire” have also been developed to realize the high resolution analysis of signal redox molecules, cells and nanoparticle mixtures.^80^–^82^

Besides target recognition, many experiments have at the same time verified the possibility that the current blockage amplitude and duration time are relevant to the 3D-structure or physical properties of a macromolecule. As shown in several recent breakthroughs,^64^,^83^–^88^ for example, solid-state nanopores were able to recognize randomly-folded or man-made structures, *e.g.*, knots^83^ and coded nanostructures^84^ in DNA, or even accurately distinguish two DNA origami structures with different shapes.^64^ In one of our recent studies, solid-state nanopores helped to distinguish twin DNA structure analogues.^89^ Increasing evidence indicates that a combination of solid-state nanopores and DNA assembly is creating a new detection methodology that may enrich DNA assembly characterization and, at the same time, extend solid-state nanopore applications.

**Fig. 1:**
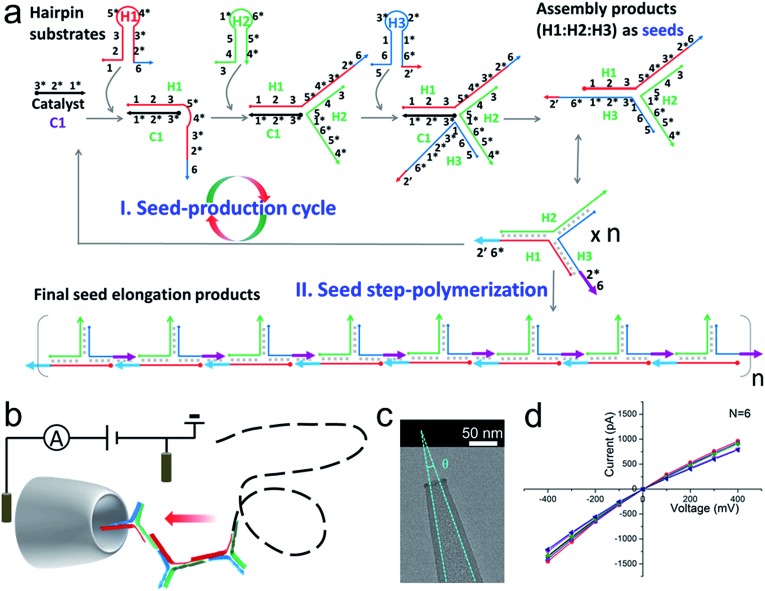
(a) A schematic diagram of SP-CHA. According to common rules, each oligonucleotide component is separated into several domains labelled with numbers. Complementarity between numbered domains is denoted by an asterisk (*). Each domain may contain 4–10 bases. Arrows denote the 3′ end of an oligonucleotide. (b) A schematic diagram of the CGN detection of SP-CHA concatemers. (c) A TEM image of a CGN. (d) The *I*–*V* characteristics of a CGN in 0.1 M KCl (*N* = 6).

Other than distinguishing structures, in this research we innovatively attend to adapting solid-state nanopores to be an effective assistant methodology to analyze catalytic amplification assembly, which usually generates complicated mixtures and thus creates a tougher task for either traditional characterization or nanopores themselves. A step polymerization based on catalytic hairpin assembly (SP-CHA), producing three-way-DNA concatemers, is self-designed as a model (Fig. 1a and b). Through counting and integrating the translocation-induced current blocks when each concatemer passes through a conical glass nanopore (CGN), we propose a quantitative method that can comprehensively profile “base-pair distribution” and “trigger-specific” signals related to SP-CHA concatemer mixtures. Because the detection takes place in a completely homogeneous solution, the results are supposed to more closely reflect the natural morphologies of the concatemers. This also helps to reveal a number of super long concatemers that were previously shown as ambiguous bands or completely undetectable by gel electrophoresis. These ultra-concatemers, longer than 2000 bp, could provide much enhanced signal-to-noise ratios for nanopores, and are thus believed to be more accurate indicators for the existence of triggers, which may benefit their use in further applications such as molecular machines or biosensors.

## Results and discussion

### The principle and verification of step polymerization based on catalytic hairpin assembly (SP-CHA)

A schematic diagram demonstrating the concept for detecting SP-CHA products using a CGN is shown in Fig. 1b. As shown in the assembly principle (Fig. 1a), SP-CHA involves two amplification pathways, seed generation and seed elongation. The seed generation process is actually a catalytic hairpin assembly reaction for three hairpin substrates (H1, H2, and H3). Being limited by a kinetic trap, assembly between the three hairpin substrates is too slow to happen. Only if a 24-mer single stranded oligonucleotide (C1) opens the first hairpin substrate (H1) through a toehold mediated strand displacement reaction will this process trigger two downstream strand displacement reactions and successively open H2 and H3. Finally, a three-way H1:H2:H3 assembly intermediate is formed. At the same time, C1 is released into the solution and can be reused as a catalyst to trigger one more assembly reaction. The success and amplifiable function of this seed production step was verified with standard fluorescence detection (Fig. 3a and b). When the H1:H2:H3 intermediate forms, it has two 16-base sticky ends (domain 2*-6 in H1, and domain 6*-2′ in H3) long enough to hybridize with each other. Therefore, it can start self-elongation as a seed and finally form concatemer products of different lengths. The success and amplifiable function of this seed elongation process could be verified through AFM imaging (Fig. 3c) and ladder-like bends are observed in an agarose electrophoresis image (Fig. 3d).

### Nanopore set-up and the verification of the event charge deficit (ECD) method for dsDNA markers and marker mixtures

**Fig. 2:**
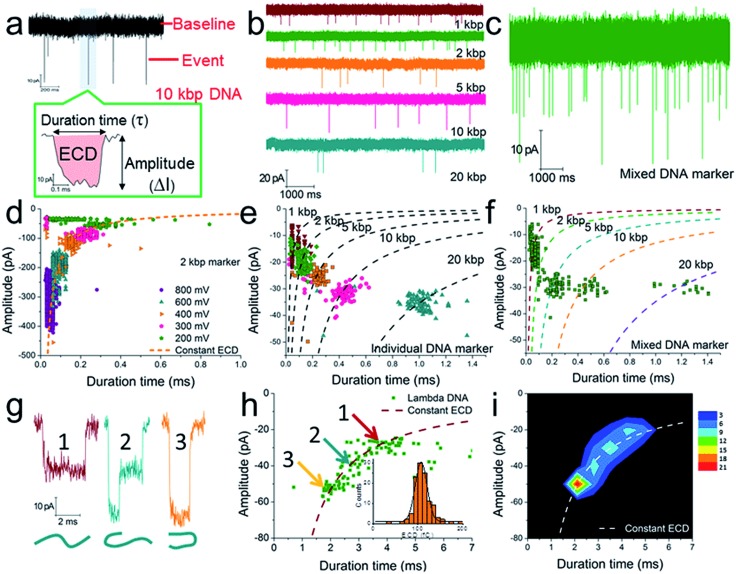
The verification of quantitative measurements of dsDNA species using a naked conical glass nanopore (CGN). (a) The current trace of a 10 kbp dsDNA marker at 300 mV. The event in the coloured area is shown with greater time resolution and with the event charge deficit (ECD) marked in red (the integrated area of the event relative to the baseline). The current trace of an individual DNA marker (b) and a mixed DNA marker (c) at 200 mV. (d) A scatter plot of amplitude *vs.* duration time for 2 kbp dsDNA marker translocation through a CGN at different voltages. (e) A scatter plot of individual dsDNA markers ranging from 1 to 20 kbp. (f) A scatter plot of mixed dsDNA markers ranging from 1 to 20 kbp (the dashed lines show the dsDNA markers fitted according to the constant ECD method, ref. 62). Note: the experiments in (e) and (f) were performed with the same pore. (g) Three characteristic current signals of lambda DNA translocation. (h and i) A scatter plot (h) and event density plot (i) showing the amplitude and duration time for lambda DNA. The inset of (h) shows a histogram of the calculated ECD values for lambda DNA. All experiments were carried out at 200 mV unless otherwise indicated. The numbers of events in each scatter plot and histogram are shown in Table S2.†

Considering that the effective diameter of SP-CHA concatemers should be close to, but larger than, the dsDNA size, CGNs of 13.1 ± 1.3 nm (mean ± SD) were specially selected for further nanopore testing, according to our previous experience.^85^ Before executing SP-CHA detection, the properties and performance of CGNs of such size were systematically tested. Their diameters were verified *via* TEM imaging (Fig. 1c) and characterized *via* typical current–voltage (*I*–*V*) curves in 0.1 M KCl electrolyte (Fig. 1d). Multiple experiments were carried out to demonstrate the good fabrication reproducibility and signaling stability, because these parameters are extremely important for getting quantitative or effective conclusions (Fig. S1–S4†). In order to systematically study SP-CHA concatemers in their original chemical environment, subsequent experiments were carried out in SP-CHA reaction buffer (500 mM LiCl, 20 mM Tris–HCl, 140 mM NaCl, 5 mM KCl and 5 mM MgCl_2_ at pH = 7.5). Fig. 2a proves that a well-accepted definite current blockage could be observed under these buffer conditions.

According to previous studies,^60^,^62^,^63^,^90^ increasing evidence indicates that a single dsDNA length can't be uniquely identified through the duration time for dsDNA translocation. It must actually be determined *via* multiple factors, including at least both the DNA length and the folding configuration of the DNA. However, a potential strategy has been proposed, which suggests that the integral of the current with respect to time, or the event charge deficit (ECD),^62^ may be more rationally used to distinguish each DNA length.^60^,^62^,^63^,^90^ In other words, regardless of how identical molecules are folded, each blocks the same amount of ionic charge movement through the pore during the total time it takes each molecule to move through the pore.^62^ The practicability of this strategy is further verified under our experimental conditions. As shown in Fig. 2d, the ECD strategy perfectly fits the amplitude-duration scatter plots for 2 kbp dsDNA markers at various voltages. The dashed orange line is fitted according to the equation ECD = –Δ*Iτ*, where Δ*I* is the current block amplitude and *τ* is the duration time of a translocation event. More dsDNA markers fitted *via* the ECD strategy are shown in Fig. S2 and S3.† All these data indicate that the ECD is a voltage-independent analysis method that is only related to the number of DNA base pairs.

When we plotted dsDNA markers ranging from 1 kbp to 20 kbp at a fixed voltage (200 mV), a very distinct event population was obtained for each marker (Fig. 2b, e and S4a–c†). This indicates that a nanopore has sufficient resolution to identify these dsDNA markers with different base pair numbers (Fig. 2e). Then an EDC fitting curve was plotted for each marker (black dashed curves in Fig. 2e). These curves can serve as standards to indicate unknown DNA species, *e.g.*, mixed DNA markers (Fig. 2c, f and S4d–f†) or further SP-CHA concatemers. Each curve represents a fixed base pair number, regardless of the folding and diameter situation. The accuracy of the ECD strategy for fitting the distribution of DNA translocations was further confirmed through experiments involving lambda DNA (48 502 bp) passing through a CGN (Fig. 2g–i). Characteristic current signals of events from the corresponding regions of the scatter plot (Fig. 2h) are shown in Fig. 2g. The event density plot (Fig. 2i) indicates that the longer DNA molecules show a broader distribution in the hyperbolae curve of ECD fitting and present a high population in folded states during translocation of the pore.

The above experiments demonstrate that the CGN method can be compared to gel electrophoresis for measuring the distribution of DNA, being especially well functioning for DNA species ranging from 1–20 kbp (the maximum limit can be pushed to nearly 50 000 bp in the form of lambda DNA, Fig. 2g–i and S3†).

### Nanopore characterization of SP-CHA concatemers

**Fig. 3:**
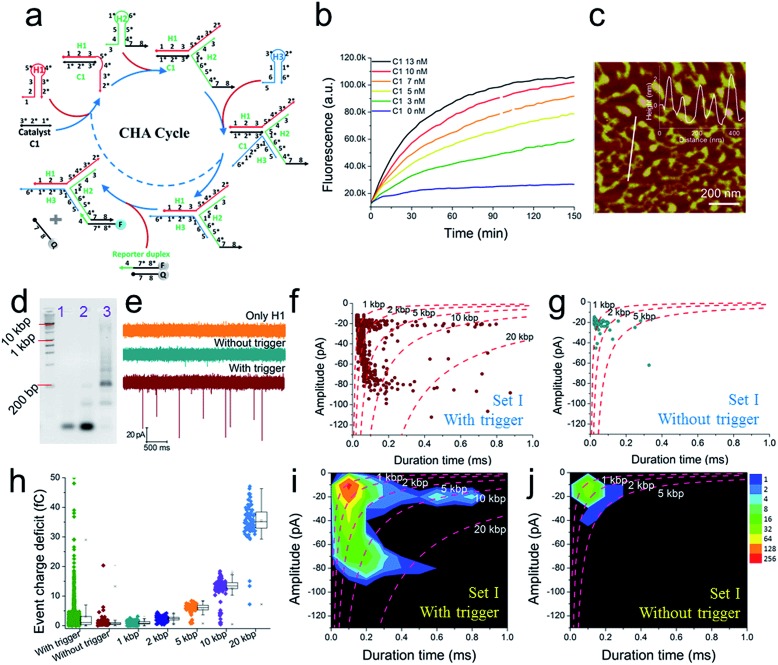
The fluorescence characterization of three-way-CHA and the characterization of step polymerization induced by catalytic hairpin assembly (SP-CHA) with a CGN. (a) A schematic diagram of a three-way-CHA pathway (seed production step) coupled with a fluorescent label. (b) Fluorescent kinetic curves of three-way-CHA (Set I) at 25 °C with different amounts of C1. The reaction included 100 nM H1, 100 nM H2, 100 nM H3 and 150 nM F–Q reporter duplex, with and without trigger C1. In the reporter duplex, [F] was 150 nM and [Q] was 200 nM. (c) An AFM image of SP-CHA concatemers; inset: cross-section analysis. (d) A 2% agarose gel electrophoresis image of SP-CHA concatemers. Lane 1: only H1; lane 2: without trigger C1; lane 3: with trigger C1. (e) The current traces of SP-CHA products with and without trigger C1. Scatter plots of SP-CHA concatemers with (f) and without (g) trigger C1 at 200 mV. (h) An ECD box chart of SP-CHA concatemers. Each box represents the 1st and 3rd quartiles of the data. The horizontal line is the median value. The square point aligned with the box shows the mean value. The whiskers extend to data points that are within 1.5× IQR. The symbol “*X*” shows data points that are within 1% to 99%. The symbol “-” shows the minimum value and maximum value. (i and j) Event density plots of SP-CHA with (f) and without (g) trigger C1. (The dashed lines show dsDNA markers fitted according to the constant ECD method, ref. 62). Note: unless otherwise indicated, all experiments were performed with SP-CHA (Set I). And the concentrations of H1, H2 and H3 were all 1 μM, which was 10 times that of C1. Four kinds of SP-CHA products were studied with the same pore in this figure and Fig. 4.

Further tests were chosen to be conducted on SP-CHA, with or without a trigger. Through counting and profiling the translocation-induced current blockage of assembly products passing through a CGN, a nanopore can provide electrophoresis-like information that clearly reflects the leakage (background signal) and degree of polymerization induced by the trigger. As presented in Fig. 3e, the CGN was too inert to sense the translocation of short hairpin substrate DNA (*e.g.*, only H1, 67 bases), merely showing a relatively steady-state background ionic current during the entire recording window. Once 0.1 μM trigger (0.1× H1) was introduced, dense and sharp “needle-like” ionic current drops appeared with respect to the baseline. In comparison, the control reaction without a trigger also displays observable ionic current blockages within the same time window, but at a lower density and current amplitude. These raw data threw light on the fact that SP-CHA reactions both with (catalytic reaction) and without (background leakage) trigger generate products bulky enough to be recognized by the CGN platform. But the catalytic reaction is much more efficient compared with leakage. These results are consistent with gel electrophoresis (Fig. 3d). Then, more quantitative information relevant to the assembly was displayed through different data presentation methods. Both scatter plots (Fig. 3f and g) and event density plots (Fig. 3i and j) are able to show the product base pair (length) distribution, with and without trigger (the dashed lines were fitted *via* the ECD constant for different DNA markers). This two-dimensional statistical chart intuitively presents the distribution trend for all events in the recording time range. Box charts of the ECD (Fig. 3h) present an electrophoresis gel-like picture, showing that trigger induced SP-CHA products have a more significant polymerization degree.

Because the nanopore used in this paper has a higher sensitivity to DNA over 1 kbp, it suitably confirms and reveals the existence of ultra-concatemers of over 10 kbp (about 3.4 μm in length), which are usually considered as smears or confusing bands in gel electrophoresis. Excitingly, we happen to scan some super long species under AFM (Fig. S5a–c†) as well, which helps to verify the production of ultra-concatemers. Correspondingly, under a high electric field driving force of 700 mV, plenty of long duration time events are observed (Fig. S5d–f†). This strong electric-field driven long concatemer translocation behaviour is consistent with previous literature reports.^85^

As shown above, unmodified bare solid-state nanopores show good reproducibility and practicability and hold special priority for discovering long and rare species. However, a pore of such size may sacrifice some resolution, so its ability to recognize small targets has usually been challenged (DNA of less than 500 bp could not be detected in this paper). Now, through these DNA assembly reactions, a small target could be transduced into a huge polymer longer than 2000 bp. These ultra-molecules can generate much more significant signals and thus serve as covalent bond-free labels that accurately indicate the existence of a trigger molecule in a homogeneous solution.

**Fig. 4:**
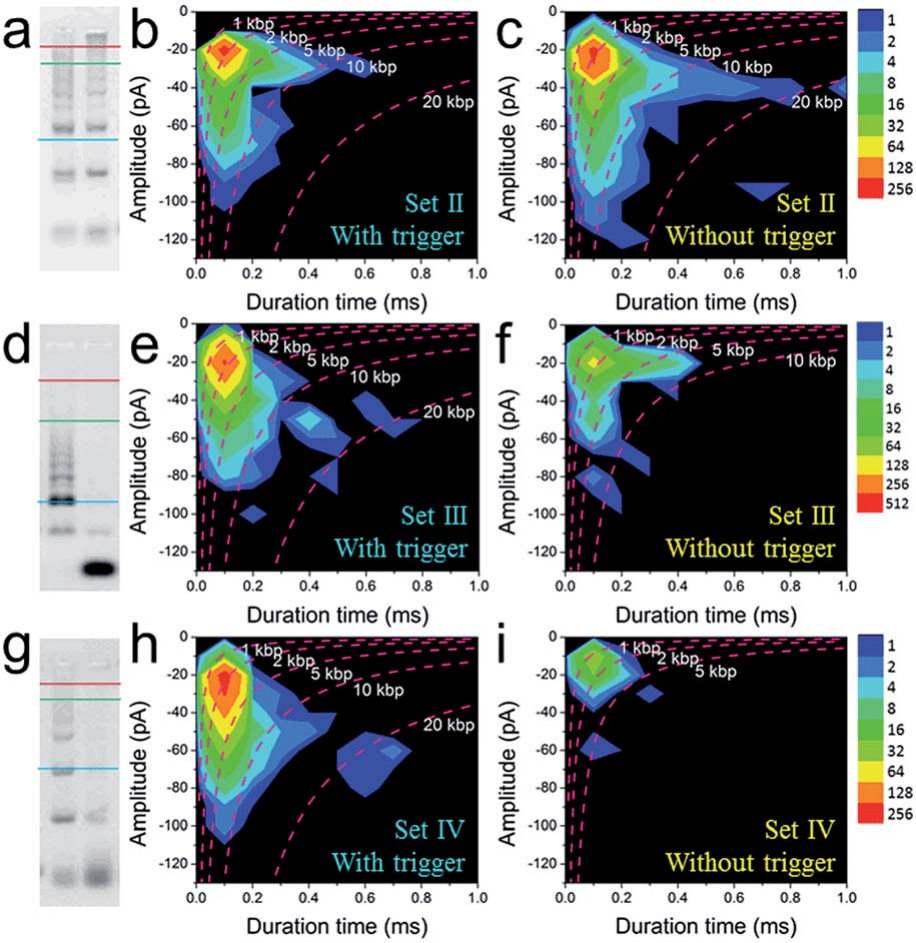
The quantitative optimization of the signal and background responses of different sets of SP-CHA assembly using CGN detection. 2% agarose gel electrophoresis images of SP-CHA-II (a), SP-CHA-III (d) and SP-CHA-IV (g). Left lane: with trigger C1; right lane: without trigger C1. Event density plots for SP-CHA-II (b), SP-CHA-III (e) and SP-CHA-IV (h) with trigger C1. Event density plots for SP-CHA-II (c), SP-CHA-III (f) and SP-CHA-IV (i) without trigger C1. For each gel picture, the blue, green, and red lines respectively represent the positions of 200 bp, 1 kb, and 10 kb dsDNA markers.

To further explore the bio-sensing ability of the current method, we paid close attention to the SP-CHA performance and the ability of the nanopore to probe subtle differences in performance. Together with the SP-CHA sequences presented above, four sets of SP-CHA components with a slight amount of change in their DNA sequences were compared (Table S1 in the ESI†). As shown in Fig. 3 and 4, event density plots can reflect the contrast between the catalytic reaction and leakage for each set of SP-CHA components, matching the gel electrophoresis pictures well. From a comparison, Set I used in Fig. 3 holds the lowest leakage and thus is selected for further concentration dependent experiments involving C1.

### The nanopore characterization of SP-CHA concatemers triggered by different concentrations of C1

**Fig. 5:**
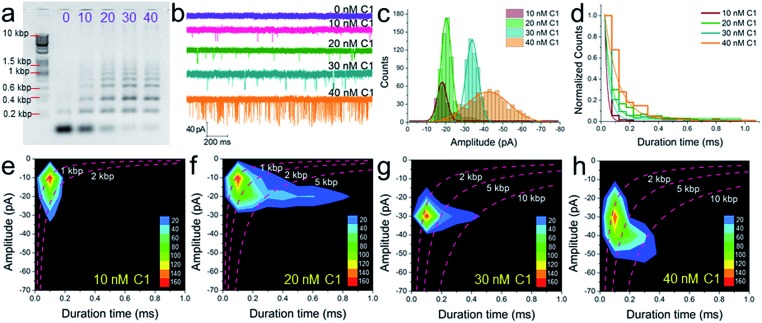
The concentration dependence of SP-CHA using CGN detection. A 2% agarose gel electrophoresis image (a), current trace (b), current amplitude histogram (c) and duration time histogram (d) of SP-CHA (Set I) with different amounts of trigger C1. (e–h) Event density plots of SP-CHA (Set I) with 10 nM (e), 20 nM (f), 30 nM (g) and 40 nM (h) trigger C1. Here, the concentrations of H1, H2, and H3 were all 300 nM. Note: the concentration dependence of SP-CHA (Set I) using CGN detection was studied using the same pore.

The conclusion demonstrated above could also be extended to other concentrations of C1 and substrates (H1, H2, and H3). For instance, under different concentrations of C1 in the presence of 300 nM H1, H2, and H3, the gel pictures only exhibit the different degrees of substrate consumption (Fig. 5a). The base pair numbers and folding conformations of the concatemers seem to be too close to be distinguished. However, nanopore detection provides more sufficient and refined information. The raw data (Fig. 5b), current amplitude histogram (Fig. 5c) and duration time histogram (Fig. 5d), respectively, show increasing translocation frequency, increasing current blockage amplitude, and increasing duration time as more and more C1 is introduced (details as shown in Table S3†). These figures can be used to reflect the C1-dependent differences in concatemer concentrations, length distributions, or even conformation variations. It should be noted that there is almost no leakage (or translocation events) observed in the sample without trigger (Fig. 5b, 0 nM C1) because the concentrations of substrates (H1, H2, H3) were much lower than those used in Fig. 3e. More importantly, the event density plots (Fig. 5e–h) also exhibited very different distributions in the presence of different amounts of C1. This indicates that the nanopore may detect concentration not only *via* translocation event frequency, but also through “fingerprint-like” patterns of scatter plots or event density plots. Here the differences in distributions are supposed to represent differences in either folding conformation, base number, or both.

## Experimental

### Materials

All chemicals were of analytical grade and purchased from Sangon Biotech (Shanghai, China) unless otherwise indicated. All oligonucleotides were ordered from Sangon Biotech (Shanghai, China) and self-PAGE purified. The oligonucleotide sequences are summarized in Table S1.† All oligonucleotides were stored in 1× TE (pH 7.5) at –20 °C.

### Fabrication of the nanopores

Conical glass nanopores were made from quartz glass capillaries (O.D: 1 mm; I.D: 0.7 mm; QF100-70-10; Sutter Instrument Co.). All glass capillaries used in the experiments were thoroughly cleaned by immersing them in freshly prepared piranha solution (3 : 1 98% H_2_SO_4_/30% H_2_O_2_) for ∼2 h to remove organic impurities. (Caution: piranha solution is a powerful oxidizing agent and reacts violently with organic compounds. It should be handled with extreme care). The capillaries were rinsed thoroughly with deionized water and vacuum dried at 70 °C prior to use. Glass nanopores were then fabricated using a CO_2_-laser-actuated pipette puller (model: P-2000, Sutter Instrument Co.) with an on-line program involving the following parameters: heat = 760; fil = 4; vel = 31; del = 120; pul = 170. TEM images were obtained using an FEI TECNAI F20 EM with an accelerating voltage of 200 kV. The tip (∼2 mm) of a nanopipette was cut off and transferred to a copper grid for TEM imaging.

### Data collection and analysis

For DNA translocation, the nanopores were assembled into homemade horizontal type glass cells (Fig. S1a†). The cell acted as the cis reservoir and the inner cavity of the glass capillary nanopore acted as the trans reservoir. Two chlorinated silver electrodes were placed in each reservoir. A potential was applied to the electrode inside the nanopore. A DNA sample was added to the cis reservoir (outside of the nanopore tip), which was set as the electrical ground. The current signal was measured using an Axopatch 200B low-noise current amplifier (Axon Instruments, USA) operating in resistive feedback mode with *β* = 1 and output gain *α* = 2. Data was low-pass-filtered at 10 kHz using the built-in 8-pole Bessel filter. The output signal was sent to a Digidata 1550B data-acquisition module (Axon Instruments, USA), collected at 100 kHz and recorded using pClamp 10.6 software. The RMS value of the measurements in this experiment is less than 2 pA. The DNA sample (in SP-CHA reaction buffer: 500 mM LiCl; 20 mM Tris–HCl; 140 mM NaCl; 5 mM KCl and 5 mM MgCl_2_ at pH = 7.5) was added into the cis reservoir. All experiments were performed at 25 °C. Translocation data were collected using a threshold, with a minimum current amplitude level of 10 pA and a minimum duration time of 0.02 ms.

### The translocation of individual and mixed dsDNA markers

DNA samples were made by diluting dsDNA markers (Nolimits chromatography-purified DNA) purchased from Thermofisher Scientific. All samples were diluted in SP-CHA buffer (500 mM LiCl; 20 mM Tris–HCl; 140 mM NaCl; 5 mM KCl and 5 mM MgCl_2_ at pH = 7.5). For the experiments shown in Fig. 1, 2 and S2–S4,† DNA markers were diluted/mixed to the same final concentration of 3.0 nM before being added to the cis reservoir. All DNA marker translocation experiments were performed with the same pore at 25 °C. The pore was rinsed with voltage-driven (bias: 600 mV; direction: cis reservoir to trans reservoir) buffer electrolyte for 5 min between each sample test.

### Experiments involving the SP-CHA reaction and agarose gel electrophoresis

For these SP-CHA reactions, stock solutions of C1, H1, H2, and H3 were diluted in 1× TNaK (20 mM Tris–HCl; 140 mM NaCl; 5 mM KCl; 5 mM MgCl_2_, pH 7.5) buffer to 10 μM. H1, H2 and H3 were then respectively annealed at 95 °C for 10 min and cooled down to 25 °C at a rate of 0.1 °C s^–1^ before use. To start the reaction, these stock solutions were diluted at suitable concentrations. 10 μL of C1, 10 μL of H1, 10 μL of H2, 10 μL of H3 and 10 μL of 2.5 M LiCl were mixed together, forming a standard 50 μL of reaction liquid. The final buffer condition was called SP-CHA buffer, and was composed of 500 mM LiCl, 20 mM Tris–HCl, 140 mM NaCl, 5 mM KCl and 5 mM MgCl_2_ at pH 7.5. After the liquid was incubated at 25 °C for at least 5.5 h, 33 μL of reaction product was used for nanopore detection, while 7 μL of it was loaded into agarose gel for electrophoresis detection. The 2% agarose gels contained 0.1 μL of Gelred per ml of gel volume and were prepared using 1× TAE buffer. Agarose gels were run at 120 V for 45 min and visualized under UV light.

### Real-time CHA fluorescence kinetic readings

All the hairpins and reporters were annealed at 95 °C for 10 min and cooled down to 25 °C at a rate of 0.1 °C s^–1^ before use. All CHA kinetic readings were performed in 1× TNaK (20 mM Tris–HCl; 140 mM NaCl; 5 mM KCl; 5 mM MgCl_2_, pH = 7.5) buffer in the presence of different volume concentrations of C1. Under both circumstances, the final SP-CHA components in the reaction mixture contained 100 nM H1 (or H2, or H3), and 150 nM reporter duplex, with or without C1. In the reporter duplex, [F] was 150 nM and [Q] was 200 nM. The fluorescence signal from 17 μL of each CHA mixture was recorded every 3 min using a Cytation-5 instrument.

## Conclusions

In this research, we report the exploration of using a solid-state nanopore to analyze an enzyme-free nucleic acid assembly (or circuit), using SP-CHA as a model. Because SP-CHA is a trigger induced reaction, it could serve as an amplifier and enhance the detection sensitivity for either the trigger itself or the nanopore. Regarding the advantages of nanopore detection, it can function like electrophoresis but at a single molecular level and in completely homogeneous systems. High sensitivity during “size-separation” also helps the discovery of long and rare species that are probably omitted by traditional characterizations. Such “pore electrophoresis” may also have unique superiority at high resolution for sensing tiny differences in 3D-folding. Therefore, it exhibits high potential as an effective characterization assistant that helps the better understanding and use of functional DNA.^37^ More importantly, using a ∼13 nm CGN and SP-CHA as a model set, we demonstrate pore-based quantitative measurements to determine the length (or base pair) distribution of both dsDNA and assembly mixtures, proposing a possible solution to increase the accuracy and fineness in unknown mixture analysis. However, it should be noted that current “quantitation curves” still have limitations. Their best functional range is from 1 kbp to 50 kbp. Once the DNA species are longer than 50 kbp, or huge enough so as to have strong interactions with the pore inner wall, the “quantitation curves” should start deviating and losing accuracy. Such limitations might be rationally addressed by adjusting the pore size, buffering conditions, and instrument parameters, just as is done in gel electrophoresis. Further comprehensive and interesting studies are needed to conclude the final deep secrets linking refined folding information with signal profiling.

## Conflicts of interest

There are no conflicts to declare.

## Supplementary Material

Supplementary information
