# Supplementary material for: Exploration of solid-state nanopores in characterizing reaction mixtures generated from a catalytic DNA assembly circuit
Source: Chem Sci. 2018 Dec 13;10(7):1953–61. doi: 10.1039/c8sc04875d (PMC6385554; doi:10.1039/c8sc04875d)
Supplement: Supplementary file 1 [file SC-010-C8SC04875D-s001.pdf]

Electronic Supplementary Material (ESI) for Chemical Science.
This journal is © The Royal Society of Chemistry 2018

Supporting Information for

Exploration of solid-state nanopore in characterizing reaction mixtures generated from catalytic DNA assembly circuit

Zhentong Zhu [†, ‡], Ruiping Wu[†, §] and Bingling Li [†,*]

†State Key Lab of Electroanalytical Chemistry, Changchun Institute of Applied Chemistry, Chinese Academy of Science, Changchun, Jilin, 130022, P.R. China.

‡University of Chinese Academy of Sciences, Beijing, 100049, China.

§University of Science and Technology of China, Hefei, Anhui 230026, China

* Email: binglingli@ciac.ac.cn. Phone: +86-431-85262008

Contents

CGN set-up and reproducibility .... Figure S1
Stability of CGN data .... Figure S2
Performance of ECD fitting .... Figure S3
Distinguish mixed DNA marker samples .... Figure S4
Characterization super long SP-CHA products .... Figure S5
Oligonucleotides used in this paper .... Table S1
Number of events in each scatter plot and histogram .... Table S2
Summary of experimental results in Figure 5 .... Table S3

1. Supporting Figures and Tables

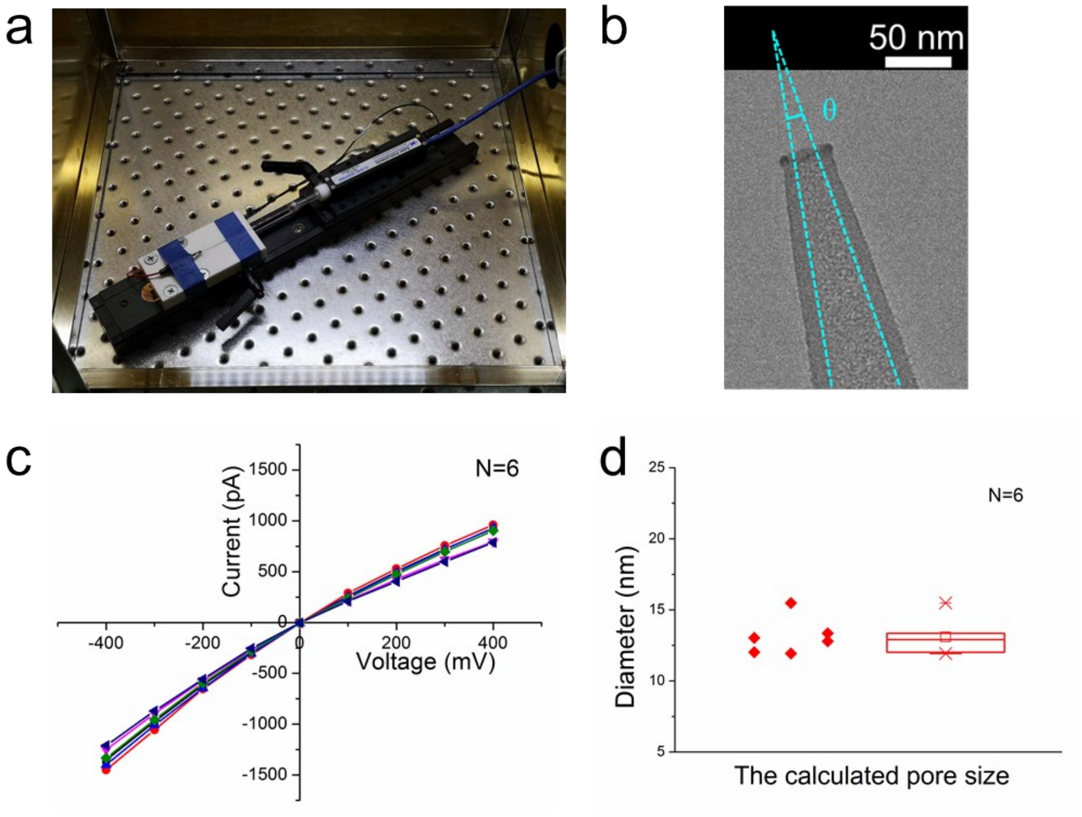

Figure S1. **CGN set-up and reproducibility.** (a) The photograph of conical glass nanopore measurement set-up. The photo was taken by one of the author of this paper. (b) TEM image of a conical glass nanopore. (c) *I-V* characteristics of conical glass nanopores in 0.1 M KCl (N=6) and corresponding calculated nanopore diameters (d). Note: Figure S1b and S1c were, in respective, identical to the Figure 1c and 1d, here to show the reproducibility of nanopore combined with the calculation method as described below.

We could use the electrochemical measurement and estimate the nanopore diameter according to the classical equation (1).[1] We chose the current data between -100mV and 100mV because in this interval the linear and rectification ratio is close to 0. The calculated pore size (13.1 nm ±1.3 nm) is in good agreement with TEM image (15 nm ± 5 nm) according to the measurements using six randomly selected pores.

$$(1): \quad a = \frac{1}{\pi \kappa R \tan\theta/2}$$

Where R is the measured pipette resistance, κ is the specific resistance of the electrolyte used (κ= 1.2 S/m in 0.1 M KCl), θ is the cone angle (θ=12 ° in Figure S1b) and a is the radius of the nanopore at the tip of the nanopipette.

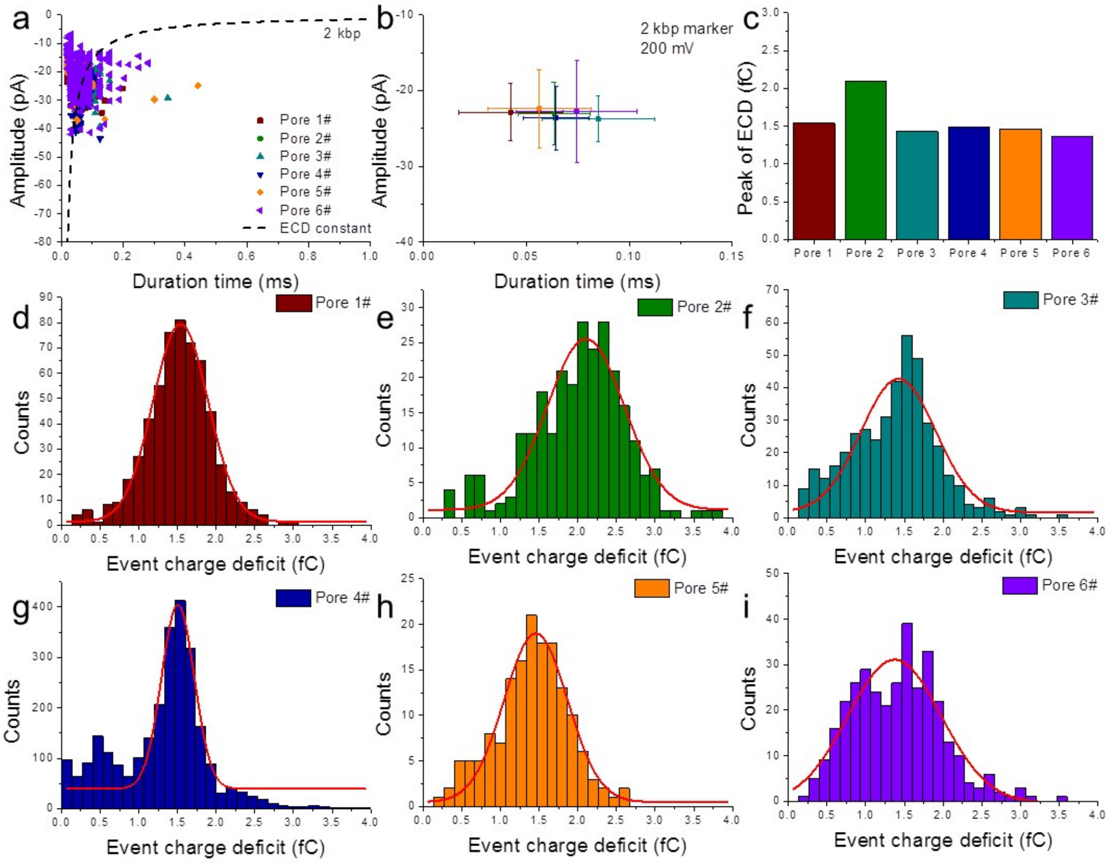

Figure S2. Stability of CGN data. Scatter plots (a) and corresponding XY error bar graph (b) of 2 kbp DNA marker at 200 mV (the measurements is used by six randomly selected pores). (c) The histogram of 2 kbp DNA marker ECD calculated in six pores, peak value of which are determined by the ECD peak location from the individual Gaussian fits (d-i). Note: Dash line in (a) was fitted by constant ECD (mean value is 1.56 fC for six randomly selected pores).

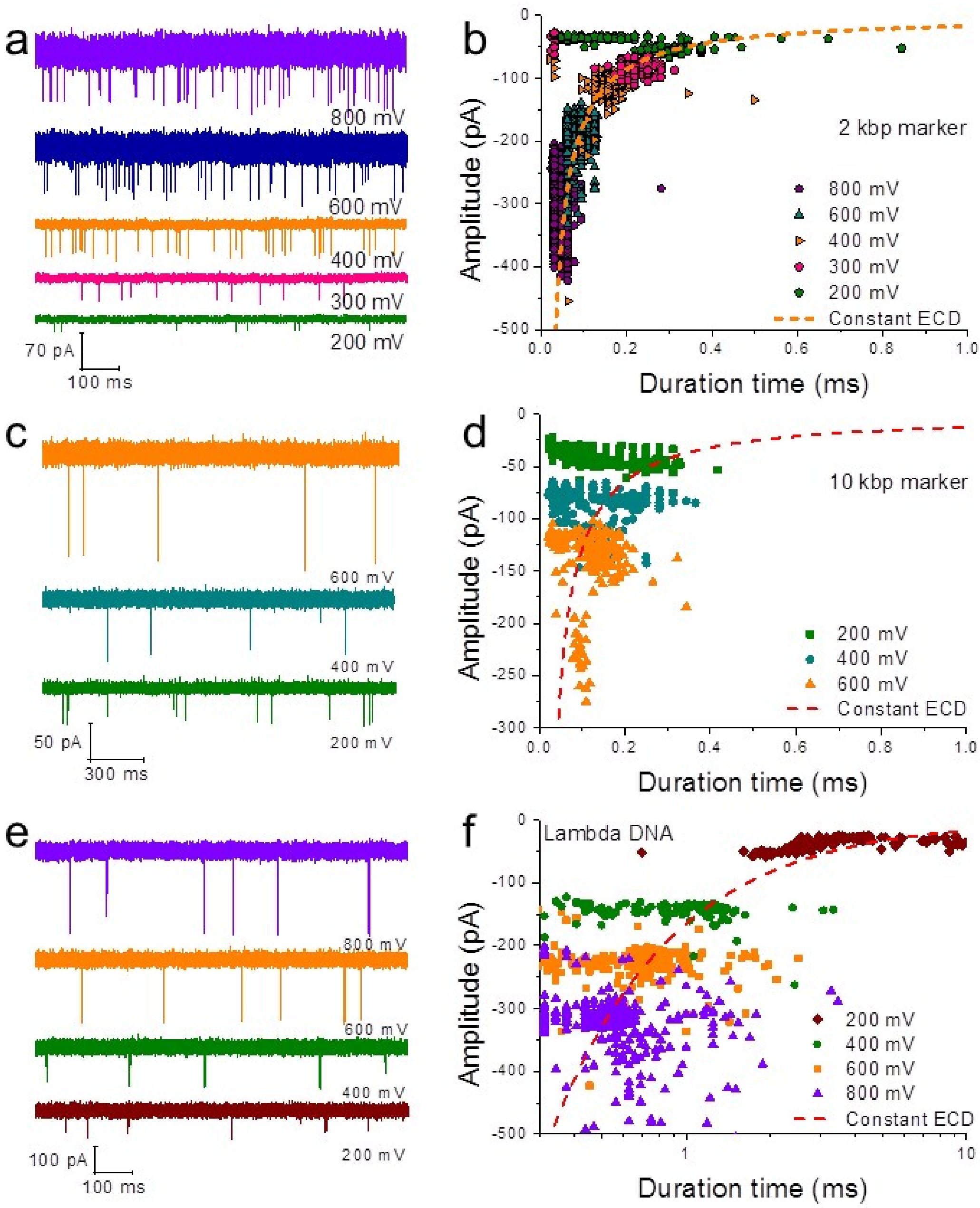

Figure S3. **Performance of ECD fitting.** Current traces recorded from 2 kbp DNA marker (a), 10 kbp DNA marker (c) and Lambda DNA (e). Scatter plots of 2 kbp DNA marker (b), 10 kbp DNA marker (d) and Lambda DNA (f). Dash lines were fitted by calculated constant ECD. Note: Figure S3b was identical to the Figure 2d, here to systematically show the voltage-independent ECD fitting strategy was perfectly matched with our experiment results. Translocation measurements in presence of different voltages were performed with the same pore in this figure.

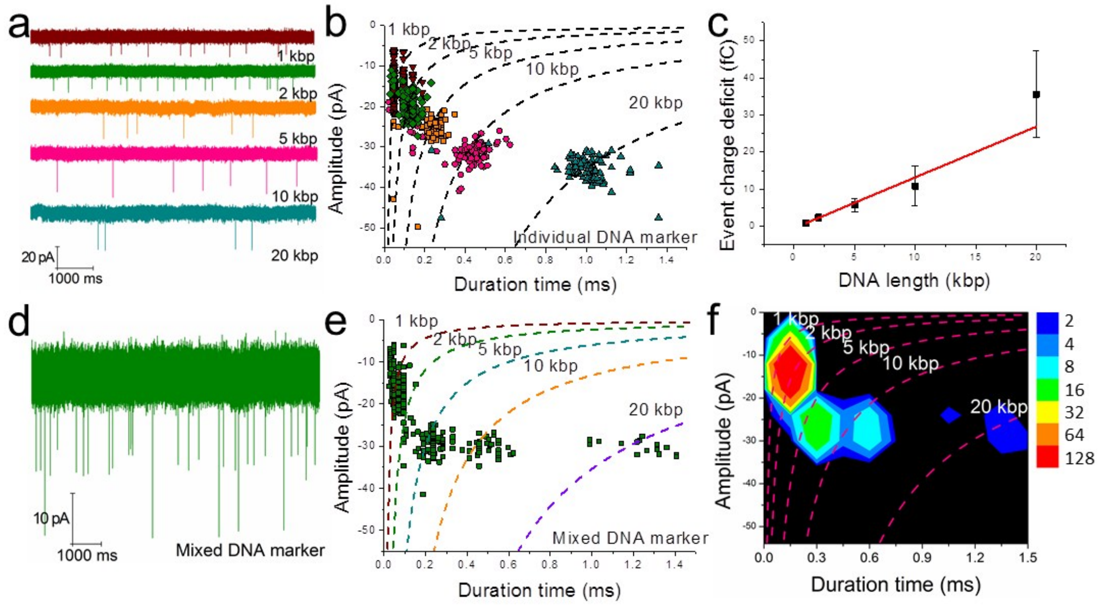

Figure S4. Distinguish of mixed DNA marker samples. Current trace of individual DNA marker (a) and mixed DNA marker (d) at 200 mV. (b) Scatter plot of individual DNA marker (length range 1 to 20 kbp) at 200 mV. (c) Calculated ECD values of DNA markers from figure S4b. (e) Scatter plot of mixed DNA marker (length range 1 to 20 kbp) at 200 mV. (d) Event density plot of mixed DNA marker. Note: All experiments as shown in this figure were performed with the same pore. Figure S4a, S4b, S4d, and S4e were, in respective, identical to the Figure 2b, 2e, 2c, and 2f here to systematically confirm the sensitivity of CGN used in this paper.

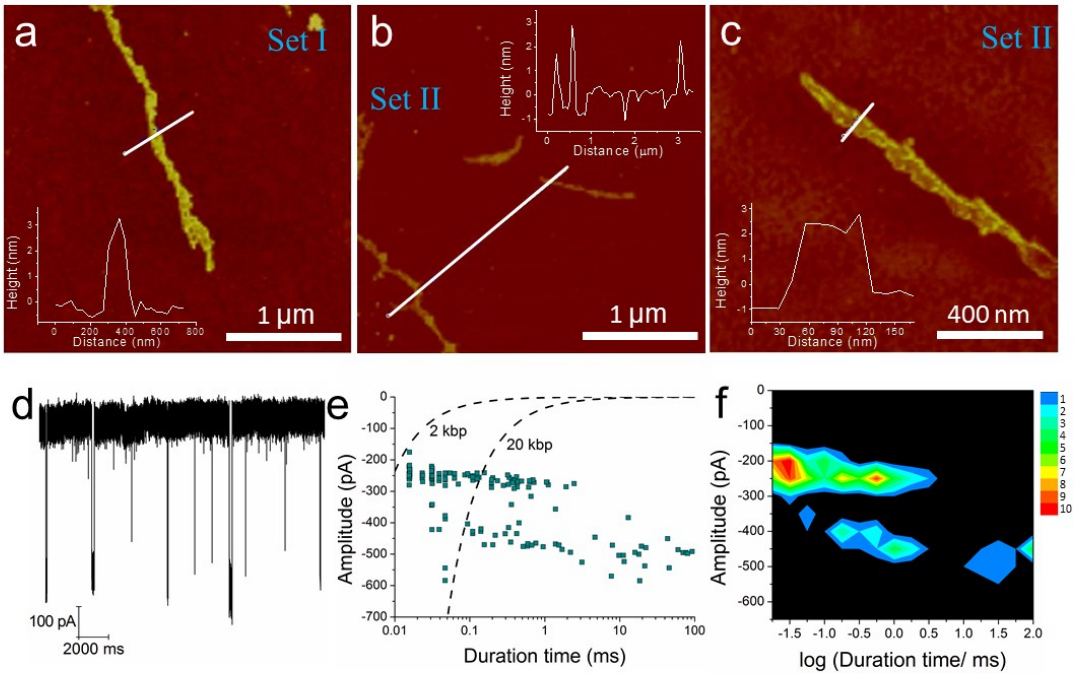

Figure S5. Characterization of Super long SP-CHA products. (a-c) AFM images of super long SP-CHA products. (d) Current traces recorded of SP-CHA (Set I) at 700 mV. (e) Scatter plot of SP-CHA (Set I) at 700 mV and corresponding event density plot (f).

<table>
<tr><th>Name</th><th>Sequence 5’-3’</th><th>Label and notes</th></tr>
<tr><td colspan="3">Sequences of four sets of SP-CHA reactions</td></tr>
<tr><td>C1</td><td>[3*|2*|1*}
[CGACATCT|AACCTAGC|TCACTGAC}</td><td>C1 is identical for all of four sets of SP-CHA</td></tr>
<tr><td>SET-1-H1</td><td>[TG|1|2|3|T|5*|4*|3*|2*|6}
TG[GTCAGTGA|GCTAGGTT|AGATGTCG|T|GAGAGAAC|CCGATATG|CGACATCT|AACCTAGC|GAGACAAG}</td><td rowspan="3">The sticky ends for seed elongation cycle are underlined.</td></tr>
<tr><td>SET-1-H2</td><td>[3|4|5|1*|6*|5*|4*|AA}
[AGATGTCG|CATATCGG|GTTCTCTC|TCACTGAC|CTTGTCTC|GAGAGAAC|CCGATATG|AA}</td></tr>
<tr><td>SET-1-H3</td><td>[5|6|1|T|3*|2*|1*|6*|2’}
[GTTCTCTC|GAGACAAG|GTCAGTGA|T|CGACATCT|AACCTAGC|TCACTGAC|CTTGTCTC|GCTAGGTTT}</td></tr>
<tr><td>SET-2-H1</td><td>[TG|1|2|3|T|5*|4*|3*|2*|6|CCTTGTCATAGAGCAC}
TG[GTCAGTGA|GCTAGGTT|AGATGTCG|T|GAGAGAAC|CCGATATG|CGACATCT|AACCTAGC/CCTTGTCATAGAGCAC}</td><td rowspan="3">The sticky ends for seed elongation cycle are underlined.</td></tr>
<tr><td>SET-2-H2</td><td>[3|4|5|1*|6*|5*|4*|AA|6’}
[AGATGTCG|CATATCGG|GTTCTCTC|TCACTGAC|CTTGTCTC|GAGAGAAC|CCGATATG|AA|GAGA}</td></tr>
<tr><td>SET-2-H3</td><td>[5|6|1|T|3*|2*|1*|6*|TT|4}
[GTTCTCTC|GAGACAAG|GTCAGTGA|T|CGACATCT|AACCTAGC|TCACTGAC|CTTGTCTC|TT|CATATCGG}</td></tr>
<tr><td>SET-3-H1</td><td>[TG|1|2|3|T|5*|4*|3*|2*)|6}
TG[GTCAGTGA|GCTAGGTT|AGATGTCG|T|GAGAGAAC|CCGATATG|CGACATCT|AACCTAGC/GAGACAAG}</td><td rowspan="3">The sticky ends for seed elongation cycle are underlined.</td></tr>
<tr><td>SET-3-H2</td><td>[3|4|5|1*|6*|5*|4*|AA}
[AGATGTCG|CATATCGG|GTTCTCTC|TCACTGAC|CTTGTCTC|GAGAGAAC|CCGATATG/AA}</td></tr>
<tr><td>SET-3-H3</td><td>[5|6|1|T|3*|2*|1*|6*|2’}
[GTTCTCTC|GAGACAAG|GTCAGTGA|T|CGACATCT|AACCTAGC|TCACTGAC|CTTGTCTC|GCTAGG}</td></tr>
<tr><td>SET-4-H1</td><td>[TG|1|2|3|T|5*|4*|3*|2*|6}
TG[GTCAGTGA|GCTAGGTT|AGATGTCG|T|GAGAGAAC|CCGATATG|CGACATCT|AACCTAGC/GAGACAAG}</td><td rowspan="3">The sticky ends for seed elongation cycle are underlined.</td></tr>
<tr><td>SET-4-H2</td><td>[3|4|5(|1*|6*|5*|4*|7|8}
[AGATGTCG|CATATCGG|GTTCTCTC|TCACTGAC|CTTGTCTC|GAGAGAAC|CCGATATG|GAGAAACA|CATCATTC}</td></tr>
<tr><td>SET-4-H3</td><td>[5|6|1|T|3*|2*|1*|6*|2’}
[GTTCTCTC|GAGACAAG|GTCAGTGA|T|CGACATCT|AACCTAGC|TCACTGAC|CTTGTCTC|GCTAGG}</td></tr>
</table>

Sequences for real-time fluorescence kinetic reading of three-way CHA cycle (Seed production)		
C1	[3*|2*|1*}<br>[CGACATCT|AACCTAGC|TCACTGAC}	
SET-1-H1	[TG|1|2|3|T|5*|4*|3*|2*|6}<br>TG[GTCAGTGA|GCTAGGTT|AGATGTCG|T|GAGAGAAC|CCGATA<br>TG|CGACATCT|AACCTAGC|GAGACAAG}	
SET-4-H2	[3|4|5|1*|6*|5*|4*|**7**|**8**}<br>[AGATGTCG|CATATCGG|GTTCTCTC|TCACTGAC|CTTGTCTC|GA<br>GAGAAC|CCGATATG|**GAGAAACA**|**CATCATTC**}	
SET-1-H3	[5|6|1|T|3*|2*|1*|6*|2'}<br>[GTTCTCTC|GAGACAAG|GTCAGTGA|T|CGACATCT|AACCTAGC| TCACTGAC|CTTGTCTC|GCTAGGTTT}	
F	[CA|8*|7*|4}<br>[CA|GAATCATG|TGTTTCTC|CATATCGG}	5' 6-FAM
Q	[A|7|8|TG}<br>[A|GAGAAACA|CATGATTC|TG}	3' BHQ1
Sequences for HCR reaction		
HCR-I	AGTCTAGGATTCGGCGTGGGTTAA	
HCR-H1	TTAACCCACGCCGAATCCTAGACTCAAAGTAGTCTAGGATTCG<br>GCGTG	
HCR-H2	AGTCTAGGATTCGGCGTGGGTTAACACGCCGAATCCTAGACTA<br>CTTTG	

Table S1: Oligonucleotides used in this paper. All labeled sequences were purified with high-pressure liquid chromatography. All unlabeled sequences were polyacrylamide gel electrophoresis purified.

	Number of events
Figure 2d	3806
Figure 2e	1282
Figure 2f	925
Figure 2h, i	453
Figure 3f, i	1109
Figure 3g, j	392
Figure 4b	1071
Figure 4c	1291
Figure 4e	1367
Figure 4f	332
Figure 4h	1097
Figure 4i	97
Figure 5c	2454
Figure 5e	323
Figure 5f	841
Figure 5g	564
Figure 5h	726
Figure S2a	4465
Figure S3d	2104
Figure S3f	2671
Figure S5e, f	371
Figure S6d, e	461

Table S2: Number of events in each scatter plot and histogram.

C1 Conc.	10 nM	20 nM	30 nM	40 nM
Event Freq.(Hz)	8	13	16	183
Amplitude (pA)	-18.1	-20.3	-33.8	-42.3
τ (ms)	0.02	0.04	0.03	0.09

Table S3: Summary of experimental results of Figure 5. Event frequency was calculated by average number of translocation events per second. The peaks in the amplitude histogram were fitted into a Gaussian to determine the mean value. The duration time histogram was fitted with an exponential function.

Reference:

(1). Fu, Y.; Tokuhisa, H.; Baker, L. A. *Chem.Commun.* **2009**, 4877.
